# Comment on: Efficacy, safety, and acceptability of misoprostol in the management of incomplete abortion: a systematic review and meta-analysis

**DOI:** 10.61622/rbgo/2025rbgo88

**Published:** 2025-10-21

**Authors:** Leidy Hernández Coronado, Jhonatan Andrés Portes Ortiz

**Affiliations:** 1 Fundación Universitaria Uninavarra Family Medicine Resident Neiva Colombia Family Medicine Resident, Fundación Universitaria Uninavarra, Neiva, Colombia.; 2 Fundación Universitaria Uninavarra Family Medicine Specialty Faculty Neiva Colombia Faculty, Family Medicine Specialty, Fundación Universitaria Uninavarra, Neiva, Colombia.; 3 Universidad El Bosque Family Medicine Bogotá Colombia Family Medicine, Universidad El Bosque, Bogotá, Colombia.; 4 Universidad Surcolombiana Clinical Epidemiology Neiva Colombia Clinical Epidemiology, Universidad Surcolombiana, Neiva, Colombia.

Dear Editor,

We read with great interest the article "Efficacy, Safety, and Acceptability of Misoprostol in the Management of Incomplete Abortion: A Systematic Review and Meta-Analysis" published on January 11, 2024 (DOI: 10.1055/s-0043-1776029),^(1)^ authored by Drs. Thiago Menezes da Silva and Moema Alves Guerra de Araujo. We wish to congratulate the authors for addressing such a relevant topic in reproductive health, particularly in contexts where access to technologies such as manual vacuum aspiration (MVA) may be limited.

Nevertheless, we consider it important to highlight certain inconsistencies in the reported results and, consequently, in the conclusions presented in the article:

1. In the results section, the authors state that 8,087 articles were identified, of which 67 duplicates were excluded. However, Figure 1 reports that 8,396 articles were retrieved, of which 67 were duplicates. Likewise, the text indicates that 7,985 articles were excluded after title and abstract screening, while the flow diagram reports 8,293 exclusions.

2. Regarding misoprostol outcomes, the authors state that the rate of complete abortion was higher in the MVA group (OR = 0.16; 95% CI = 0.07–0.36). However, figure 2 presents an OR of 0.17 (95% CI = 0.07–0.40), with the diamond favoring misoprostol—contradicting the text.

3. The text also reports that hemorrhage was more frequent in the misoprostol group (OR = 3.00; 95% CI = 1.96–4.59). Yet, Figure 3 shows that 673 of 913 patients in the MVA group experienced bleeding, compared with 581 of 924 in the misoprostol group. Thus, the bleeding rate was actually higher with MVA. Moreover, the forest plot reports an OR of 0.32 (95% CI = 0.1–1.1), crossing the null, indicating no statistical significance—again inconsistent with the text.

4. With respect to pain, the text states that pain after treatment was more frequent among patients treated with MVA (OR = 0.65; 95% CI = 0.52–0.80). However, figure 4 shows that 88 of 713 patients in the misoprostol group reported pain, compared to 33 of 699 in the MVA group, yielding an OR of 2.82 (95% CI = 1.64–4.85). This again does not match the written conclusions.

These discrepancies between the graphical data and the written statements may create confusion among readers and could potentially lead to misinterpretations in clinical practice. Given the sensitivity of this subject and the importance of evidence-based decision-making, we strongly recommend that the authors clarify these inconsistencies and explicitly explain how graphical and statistical findings were integrated into their conclusions.

We appreciate the opportunity to contribute to the academic discussion and reiterate that these observations are intended to enhance the transparency and validity of the reported results.
